# Anandamide Effects in a Streptozotocin-Induced Alzheimer’s Disease-Like Sporadic Dementia in Rats

**DOI:** 10.3389/fnins.2018.00653

**Published:** 2018-09-21

**Authors:** Daniel Moreira-Silva, Daniel C. Carrettiero, Adriele S. A. Oliveira, Samanta Rodrigues, Joyce dos Santos-Lopes, Paula M. Canas, Rodrigo A. Cunha, Maria C. Almeida, Tatiana L. Ferreira

**Affiliations:** ^1^Center for Mathematics, Computing and Cognition, Universidade Federal do ABC, São Bernardo do Campo, Brazil; ^2^Center for Natural and Human Sciences, Universidade Federal do ABC, São Bernardo do Campo, Brazil; ^3^Faculty of Medicine, University of Coimbra, Coimbra, Portugal; ^4^Center for Neuroscience and Cell Biology (CNC), University of Coimbra, Coimbra, Portugal

**Keywords:** endocannabinoid, CB_1_, HSP70, BAG2, phosphorylated tau, fear conditioning, object recognition memory, prepulse inhibition

## Abstract

Alzheimer’s disease (AD) is characterized by multiple cognitive deficits including memory and sensorimotor gating impairments as a result of neuronal and synaptic loss. The endocannabinoid system plays an important role in these deficits but little is known about its influence on the molecular mechanism regarding phosphorylated tau (p-tau) protein accumulation – one of the hallmarks of AD –, and on the density of synaptic proteins. Thus, the aim of this study was to investigate the preventive effects of anandamide (*N*-arachidonoylethanolamine, AEA) on multiple cognitive deficits and on the levels of synaptic proteins (syntaxin 1, synaptophysin and synaptosomal-associated protein, SNAP-25), cannabinoid receptor type 1 (CB_1_) and molecules related to p-tau degradation machinery (heat shock protein 70, HSP70), and Bcl2-associated athanogene (BAG2) in an AD-like sporadic dementia model in rats using intracerebroventricular (icv) injection of streptozotocin (STZ). Our hypothesis is that AEA could interact with HSP70, modulating the level of p-tau and synaptic proteins, preventing STZ-induced cognitive impairments. Thirty days after receiving bilateral icv injections of AEA or STZ or both, the cognitive performance of adult male Wistar rats was evaluated in the object recognition test, by the escape latency in the elevated plus maze (EPM), by the tone and context fear conditioning as well as in prepulse inhibition tests. Subsequently, the animals were euthanized and their brains were removed for histological analysis or for protein quantification by Western Blotting. The behavioral results showed that STZ impaired recognition, plus maze and tone fear memories but did not affect contextual fear memory and prepulse inhibition. Moreover, AEA prevented recognition and non-associative emotional memory impairments induced by STZ, but did not influence tone fear conditioning. STZ increased the brain ventricular area and this enlargement was prevented by AEA. Additionally, STZ reduced the levels of p-tau (Ser199/202) and increased p-tau (Ser396), although AEA did not affect these alterations. HSP70 was found diminished only by STZ, while BAG2 levels were decreased by STZ and AEA. Synaptophysin, syntaxin and CB_1_ receptor levels were reduced by STZ, but only syntaxin was recovered by AEA. Altogether, albeit AEA failed to modify some AD-like neurochemical alterations, it partially prevented STZ-induced cognitive impairments, changes in synaptic markers and ventricle enlargement. This study showed, for the first time, that the administration of an endocannabinoid can prevent AD-like effects induced by STZ, boosting further investigations about the modulation of endocannabinoid levels as a therapeutic approach for AD.

## Introduction

Alzheimer’s disease (AD) is characterized by multiple cognitive deficits, such as impairments of sensorimotor gating and emotional, spatial and working memory (España et al., 2010; Santos et al., 2012; Yassine et al., 2013; Cheng et al., 2014b). Besides the presence of amyloid plaques, accumulation of phosphorylated tau (p-tau) protein is one of the pathological hallmarks of AD being responsible for the destabilization of neuronal microtubules (Kadavath et al., 2015) and neuronal death (Pristerà et al., 2013). This accumulation might occur due to failures in the p-tau degradation machinery such as ubiquitin-independent proteasome pathway, which is mediated by heat shock protein 70 (HSP70) and Bcl2-associated athanogene 2 (BAG2). Although less usual, this later pathway is remarkably efficient and is disrupted during AD (Petrucelli et al., 2004; Carrettiero et al., 2009). BAG2 is also involved in several physiopathological mechanisms related to AD such as thermoregulation (de Paula et al., 2016), nicotinic receptors activation (de Oliveira et al., 2016), and the effects of Aβ_1-42_ on cell viability (Santiago et al., 2015).

HSP70 does not only play a role on tau degradation but is also involved in other mechanisms of neuroprotection, and it has been shown to interact with the endocannabinoid system. HSP70 has a high affinity for anandamide (N-arachidonoylethanolamine – AEA), functioning as a cytosolic carrier of this endocannabinoid (Oddi et al., 2009). Under pathological conditions, the enhancement of AEA levels promotes an increase in HSP70 levels and reduces dendritic loss and amyloid deposition (Tchantchou et al., 2014).

AEA (and all the endocannabinoid system) is recognized to influence the progress of AD, by regulating neurogenesis, cognitive and neuroinflammatory processes during senescence (Koppel and Davies, 2008; Marchalant et al., 2012; Bedse et al., 2014). Decreased levels of AEA have been found in the brain of AD transgenic mice and patients with AD, and were correlated with the cognitive deficits of the subjects (Jung et al., 2012; Maroof et al., 2014). Interestingly, the increase in AEA levels *in vitro* was reported to reduce tau phosphorylation through the inhibition of the activity of protein kinases (Lin et al., 2016).

In the course of AD, changes occur in the enzymatic pathways of endocannabinoids synthesis (Mulder et al., 2011) and degradation (Pascual et al., 2014) as well as in the density of cannabinoid receptor type 1 receptors (CB1R) (Farkas et al., 2012). The decreased density of CB1R, which is mostly located in synapses (Bouskila et al., 2012), is accompanied by a lower density of different synaptic markers (Canas et al., 2014), also observed in AD patients (Shimohama et al., 1997), in accordance with the hypothesis that AD begins as a synaptic dysfunction (Selkoe, 2002).

Although sporadic AD (SAD) (multifactorial AD) is the most prevalent form of the pathology, corresponding to 95% of all AD cases (Lecanu and Papadopoulos, 2013), most of the above cited studies were performed using transgenic animal models, that are more correlated to familial AD observed in humans. Deficits of non-emotional memories were widely explored using SAD models, by classical memory tasks such as the new object recognition (NOR) and Morris’ water maze (Santos et al., 2012; Yassine et al., 2013) as well as non-associative emotional memories, evaluated by the escape latency in the elevated plus maze (EPM) (Sachdeva et al., 2014). However, impairments of associative emotional memories – classical fear conditioning (España et al., 2010) and sensorimotor gating (PPI) (Cheng et al., 2014b; Koppel et al., 2014) were mostly investigated in transgenic AD animals.

Intracerebroventricular (icv) injection of streptozotocin (STZ) is a SAD model based on brain resistance to insulin (Mayer et al., 1989; Grünblatt et al., 2007; Espinosa et al., 2013; Salkovic-Petrisic et al., 2013), which mimics many of physiopathological aspects of SAD in human, like memory impairment, changes of glucose metabolism, oxidative stress and phosphorylation of tau protein (Santos et al., 2012; Peng et al., 2013; Yang et al., 2014).

The modulation of the endocannabinoid system in the development of SAD was not investigated yet, but using STZ intraperitoneally as a model for neuropathic diabetes, it was observed an increase in the hippocampal levels and activation of CB1R (Duarte et al., 2007). The endocannabinoid system seems to be involved in cerebral glucose metabolism as well, given that CB2R participate in neuronal glucose uptake, mediated by AEA (Köfalvi et al., 2016). In accordance with the tight connection between glucose metabolism and AD, which is argued to be a type 3 diabetes (De Felice, 2013; Ahmed et al., 2015), this STZ-induced AD-like sporadic dementia model seems suitable to study the endocannabinoid modulation of cognitive and molecular aspects of SAD.

Therefore, our main objective was to explore cognitive processes that are disrupted in SAD and also to investigate the effects of AEA on cognitive impairments induced by icv-STZ administration and alterations of different synaptic markers (syntaxin, synaptophysin and SNAP-25) and p-tau degradation mechanisms in the hippocampus (p-tau, HSP70, and BAG2 levels). Given the physical interaction between AEA and HSP70, our hypothesis is that AEA could modulate HSP70 levels, exerting neuroprotective effects on p-tau degradation, neuroplasticity and cognitive changes induced by STZ.

## Materials and Methods

### Animals and Drug Treatments

Male Wistar rats aged 3–4 months (350–400 g) were obtained from the Animal Experimentation Laboratory of National Institute of Pharmacology of the Federal University of São Paulo (LEA/INFARUNIFESP). Animals were housed in groups of four per cage and maintained under controlled temperature (23 ± 2°C), light/dark period of 12/12 h and with free access to food and water. All procedures were in accordance with the guidelines of the Brazilian College for Animal Experimentation (COBEA) and were approved by the Committee on Ethics in Animal Use of Federal University of ABC (CEUA–UFABC), protocol 008/13.

In our STZ-induced AD-like sporadic dementia model, rats were first anesthetized with ketamine (90 mg/kg) and xylazine (10 mg/kg) and placed in a stereotaxic apparatus. After an incision in the skin, the skull was exposed and two holes were made bilaterally, following coordinates measured from bregma (–0.8 mm on the anteroposterior, ±1.4 mm on the medial-lateral and –3.6 mm on the dorso-ventral axis, in accordance with a rat brain atlas; Paxinos and Watson, 2005).

Both STZ (Sigma) and AEA (Sigma) were diluted in citrate buffer and injected by an automated microinjection system, consisting of a microtube of polyethylene (PE-10) attached to an injection needle and to a microsyringe (Hamilton) coupled to an infusion pump (Model Bi2000 – Insight Equipment LTDA).

Each animal received two icv bilateral injections of 2 μL (1 μL/min): the first one was citrate (vehicle) or AEA (100 ng) (Fraga et al., 2009), followed by another injection of vehicle or STZ (2 mg/kg) (Motzko-Soares et al., 2018) 5 min later. After each infusion, the injection needle remained for at least 2 min in place, in order to prevent reflux of the administered solution. After surgery, animals received intramuscular injections of an antibiotic (pentabiotic, 24,000 UI/kg) and an anti-inflammatory and analgesic (meloxicam, 1 mg/kg). Also, 2 mL of saline solution were administered subcutaneously for rehydration and the animals were maintained in thermal blankets with controlled temperature until the recovery. Animals were kept in individual home cages for 5 days after surgery and body weight, food, water intake and animals well-being were accompanied daily.

**FIGURE 1:**
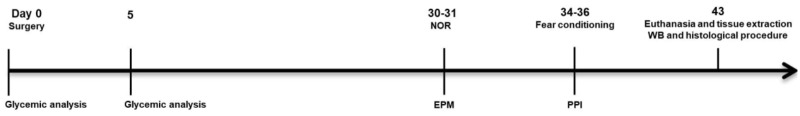
Timeline of the experimental design. Thirty days after receiving AEA (100 ng) and/or STZ (2 mg/kg), rats were submitted to behavioral tasks to detect early deficits of emotional and non-emotional memories and sensorimotor gating. One week after the end of behavioral tasks, the animals were sacrificed for either neurochemical analysis of their hippocampi by Western Blotting (WB) or histological analyses of their brains.

Before and after surgery, animals were weighted and their blood glucose was measured. Thirty days after surgery, one set of animals (*n* = 12–16 per group) was submitted to the NOR task (days 30–31), followed by contextual and tone fear conditioning (CFC and TFC, respectively) (days 34–36) and another set (*n* = 10–11 per group) was submitted to escape latency in the EPM (days 30-31), followed by pre-pulse inhibition (days 34–35). Cages and objects were always cleaned with 15% ethanol in between testing. After all behavioral tests, animals were euthanized and their brains were removed for histological or Western blotting analysis (**Figure 1**).

### New Object Recognition (NOR)

Hippocampus-dependent non-emotional memory was examined using the NOR task, using an adapted protocol after standardization tests for short-term memory evaluation (Ennaceur and Delacour, 1988; Carey et al., 2009; Botton et al., 2010; Espinosa et al., 2013). In the first phase (habituation; day 30), animals were allowed to freely explore a circular arena (60 cm in diameter) for 10 min; during training phase II (day 31), objects A and B (a pair of identical LEGO^®^ towers) were placed on opposite sides of the arena 1 cm from the walls and finally in phase III, performed 10 min later, animals returned from their home cage to the arena, where object B was replaced by a new object C (a plastic bottle) placed at the same position.

Experiments were recorded by a camera positioned on the ceiling for latter analysis of the videos. Animal’s movement throughout the arena was analyzed by Ethovision software (Noldus Information Technology Inc., Leesburg, VA, United States) and in the training and test sessions, the time that animals spent exploring each object was manually recorded. The exploration of each object was defined as the period in which the animal remained in physical and visual contact with the object (or no more than 0.5 cm far from the object), with frontal and active exploration (sniffing or manipulation, for example). The recognition index was calculated by the formula: [Time exploring object C or B/ (Time exploring object A + Time exploring object C or B)] in the training and the test sessions to evaluate the short-term recognition memory performance. In healthy animals, a higher recognition index is expected in the test compared to the training session, indicating that the animals learned the task.

### Contextual and Tone Fear Conditioning (CFC and TFC)

To evaluate associative emotional memory, animals were submitted to CFC and TFC training in conditioning box (Med-Associates, Inc., St. Albans, VT, United States), following a protocol adapted from previous studies (Blanchard and Blanchard, 1969; LeDoux, 1993; Wood and Anagnostaras, 2011; Bueno et al., 2017). The training of CFC and TFC and also the CFC test (days 34 and 35, respectively) were carried out in the context A, consisting of a conditioning box with striped walls, grid floor and bright light. TFC test (day 36) was performed in context B, a box with semicircular white walls, flat floor, light off and cleaned with acetic acid 30%. During training, after 120 s of habituation, a tone (2 kHz, 90 dB) was emitted for 30 s and in the last second, a foot-shock (1 s, 1 mA) was delivered (unconditioned stimulus-US). One minute later, the animal was placed back in its home cage.

For CFC test, animals were re-exposed 24 h after training to the context A for 240 s, without any other stimulus. During TFC test animals were placed 24 h after CFC test the context B and 2 min after habituation, the conditioned stimulus (tone) was presented twice, for 30 s after 120 s and for 30 s after 180 s (adapted from Simões et al., 2016). During all sessions, the animals’ behavior was video-taped for latter analysis using the Video Freeze software (Version 1.12.0.0, Med-Associates). The freezing time in test sessions was measured as a parameter of fear memory retention.

### Escape Latency in the Elevated Plus Maze (EPM)

The EPM used in the present study was made of white wood and consisted of a central square (10 × 10 cm) from which four arms (10 × 50 cm each) radiated outward, two of them with walls around the edge 40 cm high (closed arms), and two of them without walls (open arms). The arms were arranged so that the two open arms were opposite to each other. Arms were elevated to a height of 55 cm off the floor. The EPM is classically used for evaluation of locomotion and anxiety, but a modified version of the EPM have been also used for testing memory (Sachdeva et al., 2014; Hill et al., 2015; Mutlu et al., 2015). The experimental procedure was carried out as described by Sachdeva et al. (2014), allowing also the study of a memory parameter, measuring the retention of latency to escape to a closed arm in the test, compared to the training session.

In the training session (day 30), each animal was placed in an open arm, facing outwards and was allowed to explore the EPM for 90 s. The time taken by the rat to enter in a closed arm for the first time was measured (initial escape latency – L1) and movement parameters were recorded by a camera and analyzed by Ethovision software (Noldus Information Technology Inc., Leesburg, VA, United States). During test (day 31), animals were allowed to explore the EPM, identically as in the training session and the final escape latency was recorded (L2). The retention of escape latency [(L2/L1) × 100] represents the learning of non-associative emotional memory, related to the natural tendency of rodents to prefer dark and closed places.

### Prepulse Inhibition (PPI)

Two startle chambers were used to perform the PPI test (SR-LAB, San Diego Instruments, San Diego, CA, United States). Each box contained a loudspeaker located 24 cm above a Plexiglas cylinder fixed on a platform. The startle responses were detected within the cylinder and digitized by a piezoelectric accelerometer positioned below this platform. The startle amplitude was calculated by the mean of 100 samples during the first 100 ms after the onset of the stimulus (1/ms) and presented in arbitrary units (AU). Five days before to surgery, the animals were submitted to a PPI matching session and homogeneously distributed among the groups based on their natural percentage of PPI.

After surgery (day 34 or 35), animals were submitted to a new PPI session identical to the matching procedure (adapted from Rodrigues et al., 2017). The test session started with an acclimatization period of 5 min with a constant background white noise (60 dB). Then, the animals were exposed to various combinations of white noise pulses with random intertrial intervals of 10–20 s for 20 min. The stimuli could be one of four types: pulse alone (40 ms, 120 dB), prepulse alone (20 ms, 70, 75, or 80 dB), pulse preceded by prepulse (100 ms interval) or no stimulus, except the background noise. The different types of stimuli were presented pseudorandomly so that no combination was presented twice consecutively. The PPI percentages were calculated by the relation between the means of acoustic startle response (ASR) amplitudes to prepulse-pulse trials to the pulse alone ones. According to the formula: 100 × (ASR to pulse - ASR to prepulse-pulse)/ASR to pulse.

### Protein Extraction and Western Blotting

One week after all behavioral tests, animals randomly assigned for Western Blotting analysis were euthanized by decapitation. Their brains were quickly dissected to isolate the hippocampus, which was frozen in liquid nitrogen and stored at –80°C. For protein extraction, hippocampal tissue was homogenized, and proteins were extracted with RIPA buffer (100 mM Tris, pH 7.4, 10 mM EDTA, 10% SDS, 100 mM sodium fluoride, 10 mM sodium pyrophosphate, 10 mM sodium orthovanadate, 1 mM DTT, 1 mM PMSF, 1% protease inhibitor cocktail from Sigma-Aldrich, St. Louis, MO, United States).

The preparation of synaptosomes was carried out as previously described (Canas et al., 2009). Briefly, hippocampi were homogenized with ice-cold buffered sucrose solution (pH 7.4, 0.32 M) and then the homogenate was centrifuged at 3,000 ×*g* for 10 min at 4°C. The supernatant was collected and centrifuged at 14,000 ×*g* for 12 min at 4°C. Next, the supernatants were discarded and the pellets resuspended in 45% Percoll, before being centrifuged (16,000 ×*g*, 2 min at 4°C). The top layer was collected and resuspended in Krebs-HEPES-Ringer solution and centrifuged again (16,000 ×*g*, 2 min at 4°C). The pellet was finally resuspended in lysis buffer for subsequent analysis of synaptophysin, syntaxin-I, SNAP-25 and CB1R. The quantification of protein levels was performed using BCA colorimetric assay, using bovine serum albumin (BSA) as standard, to allow equalizing the protein concentration of all samples by adding sample buffer. Samples were then denatured for 20 min at 70°C and stored at –20°C for further use.

Total cell lysates or synaptosomes (10 or 20 μg, depending on the protein) were separated by 12% SDS-PAGE and transferred to nitrocellulose membrane. Membranes were blocked with Tris-buffered saline (TBS, 5% non-fat milk) for 1 h at 20°C, followed by incubation for 24 h at 4°C with the primary antibodies: mouse anti-total tau (1:1,000, Genway), rabbit anti-p-tau Ser199/202 (1:1,000, Sigma), rabbit anti-p-tau Ser396 (1:1,000, Abcam), rabbit anti-BAG2 (1:1,000, Novus Biologicals), rabbit anti-Hsp70 (1:1,000, Sigma), goat anti-CB1R (generously supplied by Ken Mackie, Indiana University), mouse anti-synaptophysin (1:20,000, Sigma), mouse anti-syntaxin-I (1:20,000, Sigma), mouse anti-SNAP-25 (1:20,000, Sigma), mouse anti-α-tubulin (1:10,000, Sigma) and mouse anti-β-actin antibody (1:2,000, Novus Biologicals). After washing with TBS with Tween 20 (TBS-T), membranes were incubated with the appropriate secondary antibody (1:5,000, Thermo Fisher Scientific, Waltham, MA, United States), conjugated to peroxidase, for 2 h at 20°C. Membranes were then washed in TBS-T for 20 min and incubated with chemiluminescent reagent. Quantification of the optical density of the bands was performed using Image Lab 6.0 (Bio-Rad Laboratories, Inc. United States) and values were normalized by β-actin or α-tubulin density and expressed as percentage related to control samples (Veh/Veh).

### Perfusion and Histological Procedure

Animals randomly assigned for histological analysis were overdosed with urethane and transcardially perfused initially with phosphate-buffered saline (PBS 0.01 M, pH 7.4) and then with 4% paraformaldehyde in 0.01 M PBS, pH 7.4, for fixation. After perfusion, brains were removed and stored in the same fixative solution for 24 h. Brains were then transferred to a 30% sucrose solution in 0.01 M PBS, frozen in dry ice and stored at -80°C until being sliced in a cryostat (–20°C) into 40 μm coronal sections, which were mounted on gelatinized blades and stained with cresyl violet. Slices were photographed using a light microscope (Carl Zeiss Microscopy, Thornwood, NY, United States) for the qualitative and quantitative analysis of the cross-sectional area of the lateral ventricles (LV), which was used as a general index of alteration of brain morphology, often associated with neuronal damage.

### Statistical Analysis

Treatment was considered the categorical factor and one-way ANOVA was used for statistical analysis of movement, anxiety and memory parameters in EPM and fear conditioning training as well as for the measurements of ventricle area and the densities in the Western blotting. Body weight, blood glucose levels, memory parameters in the NOR, CFC, TFC, %PPI, and ASR were analyzed by repeated measures two-way ANOVA. Data were expressed as means ± SEM and Duncan’s test was used as *post hoc* in all experiments. Differences were considered statistically significant when *p* ≤ 0.05 and all analysis were performed using Statistica 7.0 Stat Soft, Inc. 2004.

## Results

### Behavioral Analysis

#### Physiological Parameters

No differences in body weight and plasma levels of glucose were found between the different treatments, before and after surgery (data not shown). This suggests that the impact of icv-administered STZ seems restricted to the brain and does not cause evident peripheral alterations. These lack of alterations also indicate that the recovery from surgery was not affected by the treatments.

#### AEA Prevents STZ-Induced MEMORY Impairment in the NOR

**FIGURE 2:**
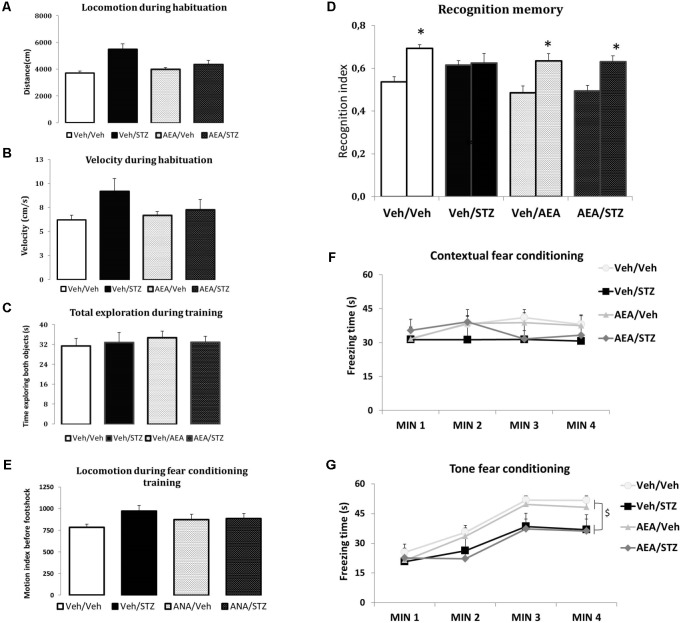
Performance of Wistar rats icv-treated with AEA and/or STZ (Veh/Veh, Veh/STZ, AEA/Veh, and AEA/STZ) in ambulatory **(A,B)**, exploratory **(C)**, and memory **(D)** parameters in the NOR task carried out 30 days after the drug treatments, and in the CFC **(E)** and TFC **(F)** 34 days after treatment. **(A)** Traveled distance (in cm) and **(B)** velocity (in cm/s) during the habituation session, represented as means ± S.E.M. (*n* = 7–10 animals per group). No differences were detected between groups (one-way ANOVA). **(C)** Total time exploring objects **(A,B)** during the training and testing phases, represented as means ± S.E.M. (*n* = 13–14 animals per group). No differences were detected between groups (repeated measures ANOVA). **(D)** Recognition index in the training (first bar of each pair) and in the test (second bar of each pair) (represented as means ± S.E.M. (*n* = 13–14 animals per group). ^∗^*p* < 0.05, training is different from test (repeated measures ANOVA, followed by Duncan’s post-test). **(E)** Shows the average motion index during 2 min of habituation in the CFC training, before the delivery of the footshock, presented as means ± S.E.M. (*n* = 12–17 animals per group). No differences were found between groups (repeated measures ANOVA). **(F)** Shows the freezing time during 4 min in the CFC test, 24 h after training, presented as means ± S.E.M. (*n* = 12–17 animals per group). No differences were found between groups (repeated measures ANOVA). **(G)** Freezing time during 4 min in the TFC test, presented as means ± S.E.M. (*n* = 12–17 animals per group). Freezing time of both groups treated with STZ was decreased. ^$^*p* < 0.05, Veh/STZ and AEA/STZ are different from Veh/Veh and AEA/Veh (repeated measures ANOVA, followed by Duncan’s post-test).

None of the treatments interfered with the distance traveled and velocity during the habituation phase or with the total time spent exploring both objects in training phase of the NOR test, indicating that AEA and/or STZ did not modify spontaneous locomotion (**Figures 2A,B**) and exploration patterns (**Figure 2C**). Considering recognition memory performance, the interaction between treatment and sessions had a significant effect [*F*_(3,51)_ = 3.5611, *p* = 0.020] and *post hoc* comparisons showed that Veh/Veh, AEA/Veh and AEA/STZ presented a higher NOR index in the test compared to the training session (*p* < 0.001 in all cases), while only Veh/STZ did not show difference between the training and the test sessions (*p* = 0.792). These results indicate that Veh/STZ animals did not learn the task and AEA prevented this cognitive impairment in AEA/STZ animals (**Figure 2D**).

#### AEA Does Not Alter STZ-Induced Deficits in Fear Memory

The measures of average motion index during the fear conditioning training showed that none of the treatments affected spontaneous locomotion before the delivery of the footshock (**Figure 2E**). The CFC test revealed no differences related to treatment, time or the interaction between treatment and time (**Figure 2F**). In the TFC test, treatment [*F*_(3_, _54)_ = 3.2241, *p* = 0.029] and time [*F*_(3_, _162)_ = 69.985, *p* < 0.001] showed significant differences. However, the interaction between these factors was not significant. *Post hoc* comparisons showed that freezing time was increased in the minutes after the tone emission compared to the period before tone (*p* < 0.001), confirming an association between the conditioned and unconditioned stimuli (**Figure 2G**). Further *post hoc* comparisons of treatment effect revealed that Veh/STZ and AEA/STZ presented less freezing time than Veh/Veh (*p* = 0.030 and *p* = 0.031, respectively) indicating that STZ disrupted memory performance and AEA was devoid of effect (**Figure 2G**).

#### AEA Partially Prevents STZ-Induced Memory Impairment in the EPM

**FIGURE 3:**
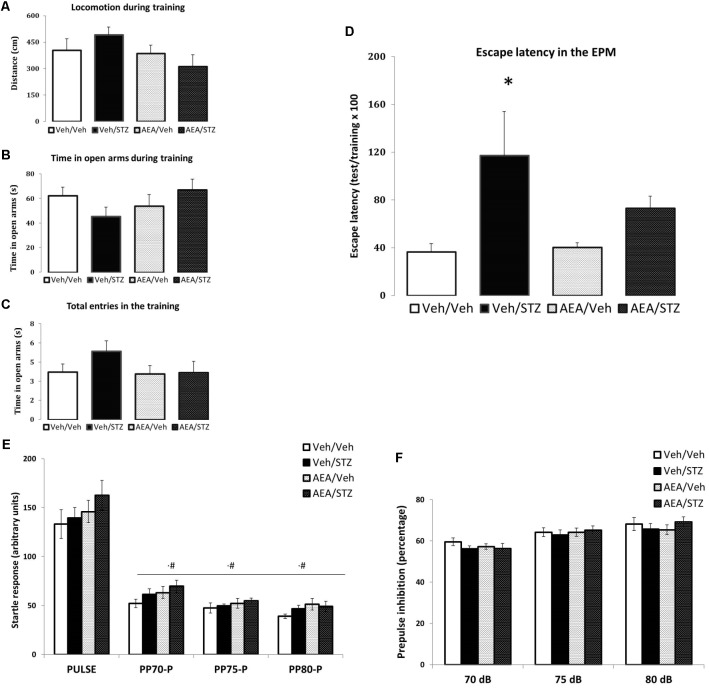
Performance of Wistar rats icv-treated with AEA and/or STZ (Veh/Veh, Veh/STZ, AEA/Veh, and AEA/STZ) in anxiety **(A–C)** and memory **(D)** parameters in the escape latency test in the EPM 30 days after treatment and in startle **(E)** and prepulse inhibition responses **(F)**. **(A)** Distance (in cm), **(B)** time spent in open arms and **(C)** the number of total entries during the training session, represented as means ± S.E.M. (*n* = 56 animals per group). No differences were detected between groups (one-way ANOVA). **(D)** Percentage of the time needed to enter in one closed arm in the test session compared to training, represented as means ± S.E.M. (*n* = 11–12 animals per group. ^∗^*p* < 0.05, Veh/STZ is different from Veh/Veh (repeated measures ANOVA, followed by Duncan’s post-test). **(E)** Startle response after a tone stimulus of 120 dB (pulse) preceded or not by a tone stimulus of 70, 75, or 80 dB (prepulse) presented in a pseudorandomic manner, represented as means ± S.E.M. (*n* = 7–10 animals per group). ^#^*p* ≤ 0.05, ASR response with pulse preceded prepulse of 70, 75, and 80 dB was lower than pulse alone (repeated measures ANOVA, followed by Duncan’s post-test). **(F)** Percentage of inhibition of startle response caused by the prepulse + pulse (prepulse inhibition) compared with the response to the pulse alone represented as means ± S.E.M. (*n* = 7–10 animals per group). No differences were found between treatments.

In the EPM test, the spontaneous ambulation measured during training session, was not altered by any treatment (**Figure 3A**) and the same occurred with anxiety-like parameters such as the time spent in the open arms (**Figure 3B**) and the number of total entries (**Figure 3C**). Treatments altered the retention of escape latency to a closed arm [*F*_(3_, _39)_ = 3.0160, *p* = 0.041] and *post hoc* comparisons showed significant higher escape latency in Veh/STZ compared to Veh/Veh (*p* = 0.021), but not to AEA/STZ (*p* = 0.166). However, AEA/STZ was not different from Veh/Veh (*p* = 0.279), suggesting that AEA partially attenuated the impairment of non-associative emotional memory caused by STZ (**Figure 3D**).

#### STZ and/or AEA Did Not Alter Sensorimotor Gating Responses in the PPI

In the PPI test, no effect of treatments on ASR was detected. Data analysis revealed differences only regarding the type of the stimulus [*F*_(3_, _90)_ = 321.11, *p* < 0.001] and *post hoc* comparisons showed lower ASR level of pulses preceded by prepulses of 70, 75, and 80 dB compared to pulse alone trials (*p* = 0.006, *p* < 0.001, and *p* < 0.001, respectively). The interaction between treatment and type of stimulus was also not significant (**Figure 3E**).

Differences in the percentage of PPI among treatments were also not significant. Yet, different intensities of prepulse were able to induce different %PPI [*F*_(2_, _60)_ = 24.308, *p* < 0.001] and *post hoc* comparisons indicated that PPI elicited by 70 dB differs from 75 and 80 dB (*p* < 0.001 in both cases) and that the PPI values between 75 and 80 dB also differ (*p* = 0.039). The interaction between treatment and prepulse intensity was not significant (**Figure 3F**). Therefore neither STZ nor AEA modified sensorimotor gating responses.

### Histological Analysis of the Area of Lateral Ventricle

**FIGURE 4:**
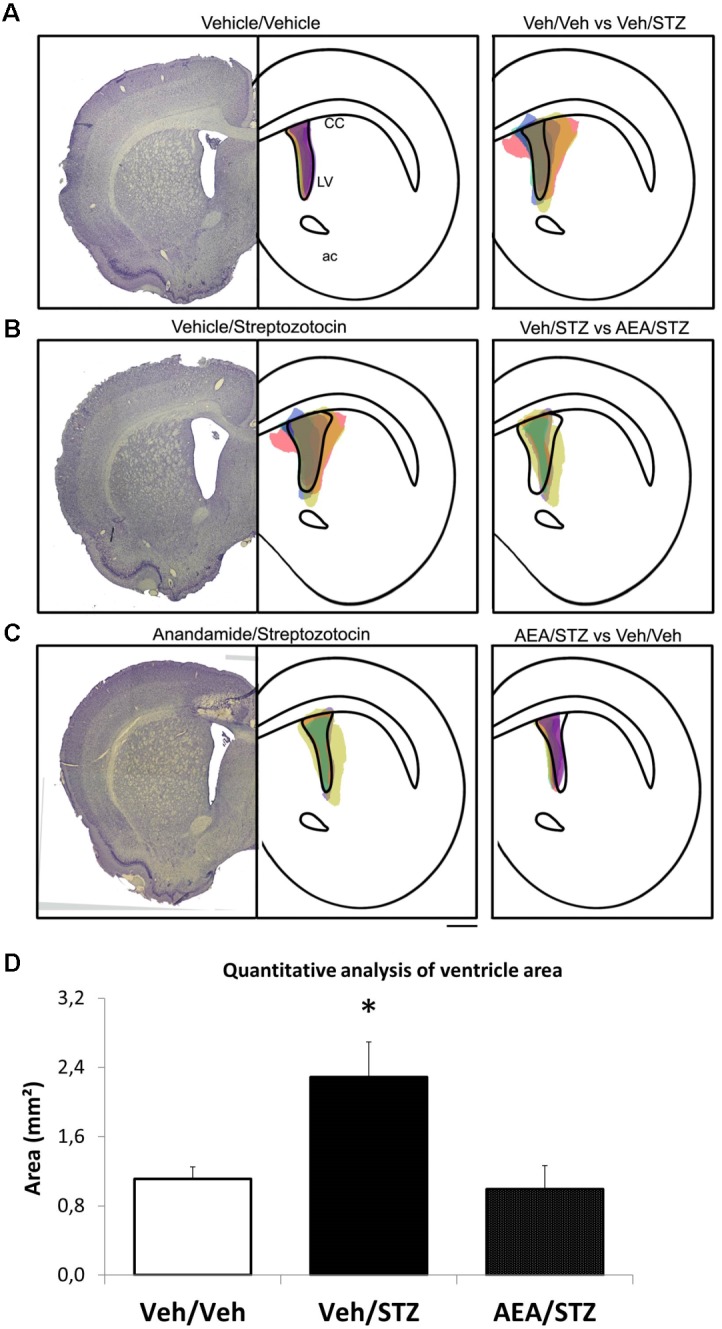
Schematic representation and quantitative analysis of the cerebral ventricular area of Veh/Veh, Veh/STZ, and AEA/STZ animals. The left panel presents a representative histologic image of the lateral ventricle (LV) of the Veh/Veh groups in **(A)**, Veh/STZ in **(B)**, and AEA/STZ in **(C)**. The central panel represents schematically the shape of the histological figure from left, filled with colored spots corresponding to the ventricular area of each of the samples used in the respective groups (*n* = 3–5). The right panel represents the central figure with the shape of the ventricle of one group overlapped with the ventricular area of the samples from another group (**A**: Veh/Veh × Veh/STZ, **B**: Veh/STZ × AEA/STZ and **C**: AEA/STZ × Veh/Veh). **(D)** shows the quantitative analysis of ventricle area (mm^2^), measured + 1.56 mm (anteroposterior) from bregma. ^∗^*p* < 0.05, area values are different from Veh/Veh and AEA/STZ (one-way ANOVA, followed by Duncan’s post-test).

The quantitative analysis of the area of the lateral ventricle (LV) (using a protocol adapted from Shoham et al., 2003) (**Figure 4**) showed an effect of the treatment [*F*_(2,9)_ = 6.3582, *p* = 0.019] and *post hoc* comparisons detected an enlargement of the LV area in Veh/STZ when compared to Veh/Veh (*p* = 0.017) and AEA/STZ (*p* = 0.014). This suggests an ability of AEA to prevent STZ-induced ventricle enlargement, which is an indicative of neuronal loss, a common feature of AD-like sporadic dementia.

### Neurochemical Analysis

#### Levels of Synaptic Proteins and CB1R

**FIGURE 5:**
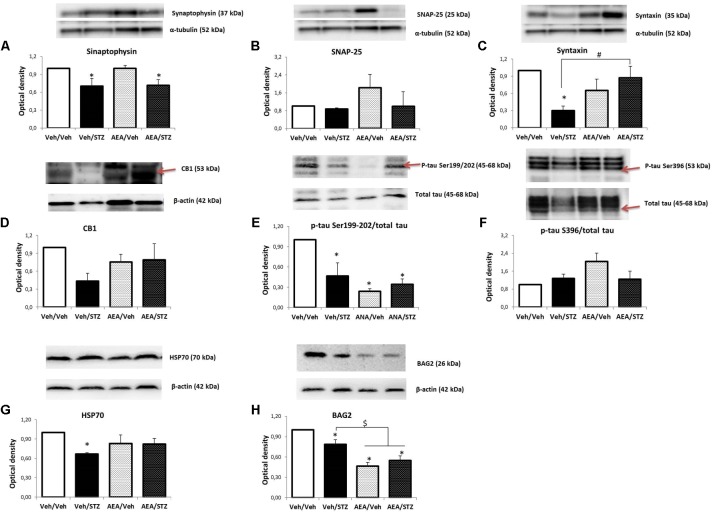
Levels of synaptophysin **(A)**, SNAP-25 **(B)**, syntaxin **(C)**, CB1 receptor **(D)**, p-tau Ser199/202/total tau **(E)**, p-tau Ser396/total tau **(F)**, HSP70 **(G)**, and BAG2 **(H)** in the hippocampus of Wistar rats icv-treated with AEA and/or STZ (Veh/Veh, Veh/STZ, AEA/Veh, and AEA/STZ), quantified by Western blotting. Synaptophysin, SNAP-25 and syntaxin levels were normalized by α-tubulin levels. CB1 receptor, HSP70 and BAG2 levels were normalized by β-actin levels. P-tau Ser199/202 and Ser396 levels were normalized by total tau levels. Data are represented as means ± S.E.M of the percentage of groups treated with AEA and/or STZ compared to Veh/Veh, which was considered 1.0 (*n* = 3-5 animals per group), ^∗^*p* ≤ 0.05, optical density values are different from Veh/Veh, ^#^*p* ≤ 0.05, optical density values of AEA/STZ are different from Veh/STZ, ^$^*p* ≤ 0.05, optical density values of AEA/Veh and AEA/STZ are different than Veh/Veh and Veh/STZ (one-way ANOVA, followed by Duncan’s post-test). Red narrows indicate the bands that were analyzed.

The levels of synaptophysin in synaptosomes were affected by treatment [*F*_(3_, _12)_ = 3.4400, *p* = 0.051] and *post hoc* comparisons revealed that Veh/STZ and AEA/STZ display lower levels than Veh/Veh (*p* = 0.045 and *p* = 0.048, respectively) (**Figure 5A**). SNAP-25 levels remained unaltered by treatments (**Figure 5B**) while syntaxin levels were reduced [*F*_(3_, _8)_ = 7.8587, *p* = 0.009] in Veh/STZ compared to Veh/Veh (*p* = 0.003). AEA/STZ rescued this depletion of syntaxin levels, presenting higher optical density values than Veh/STZ (*p* = 0.007) (**Figure 5C**). Regarding to levels of CB1R, one-way ANOVA did not detect significant differences but given that we observed a tendency for a decrease of CB1R levels only in Veh/STZ compared to Veh/Veh, an analysis with independent samples *t*-test was performed and confirmed this tendency of difference between these two groups (*p* = 0.004) and also confirmed that AEA/STZ was not different from Veh/Veh (*p* = 0.538) and from Veh/STZ (*p* = 0.379), presenting an intermediary value (**Figure 5D**).

#### Levels of p-Tau, Total Tau, HSP70, and BAG2

One-way ANOVA revealed a significant effect of treatment on the p-tau Ser199/202/total tau ratio [*F*_(3_, _8)_ = 5.8893, *p* = 0.020] and *post hoc* comparisons showed that p-tau levels were decreased in Veh/STZ, AEA/Veh, and AEA/STZ, compared to Veh/Veh (*p* = 0.027, 0.006, and 0.017, respectively) (**Figure 5E**). In contrast, p-tau Ser396/total tau ratio remained unaltered (**Figure 5F**). HSP70 levels were also affected by treatment [*F*_(3_, _8)_ = 4.3391, *p* = 0.043] only in Veh/STZ compared to Veh/Veh (*p* = 0.009) (**Figure 5G**). BAG2 levels were decreased [*F*_(3_, _8)_ = 19.412, *p* < 0.001] in Veh/STZ, AEA/Veh and AEA/STZ compared to Veh/Veh (*p* = 0.025, *p* < 0.001, and *p* < 0.001, respectively). Moreover, AEA/Veh and AEA/STZ groups displayed even lower levels of BAG2 in relation to Veh/STZ (*p* = 0.004 and *p* = 0.015, respectively), showing that AEA *per se* decreased BAG2 levels (**Figure 5H**). Please see **Supplementary Figure 1** to check complete bands of all the proteins analyzed in this study.

## Discussion

The main finding of the present study is that AEA prevented memory impairments induced by STZ in two memory tasks. These tasks, especially NOR, are commonly used to validate the behavioral impairment in STZ-treated rats. Indeed, previous studies (Espinosa et al., 2013; Bassani et al., 2017), observed deficits of NOR and object location recognition 28 days after administration of icv-STZ (3 mg/kg) with different intervals between training and test sessions. Considering the evaluation of the escape latency as a parameter of emotional memory in the EPM, Sachdeva et al. (2014) and colleagues found STZ effects similar to our study: using also an interval of 24 h between acquisition and retention sessions, animals presented greater escape latency 20 days after icv-STZ (3 mg/kg) compared to control. Moreover, no alterations were found in our study regarding locomotion and anxiety-like parameters in the open field, EPM or fear conditioning training. A tendency for an increase in several anxiety-like behaviors was observed only in the Veh/STZ group, but it did not reach statistical significance.

Although several molecular alterations of AD arise only months after STZ administration (Knezovic et al., 2015), the deterioration of some types of memory seems to emerge even earlier in this model. Indeed different studies reported an impairment of spatial working memory in the Morris’ water maze (MWM) 3 h and 15 days after icv administration of STZ (3 mg/kg) (Santos et al., 2012; Sachdeva et al., 2014). Most studies using icv-STZ generally use a dose of 3 mg/kg (Jayant et al., 2016) but we choose 2 mg/kg in order to trigger mild and non-generalized cognitive alterations. Given that we detected impairments of recognition, non-associative emotional and cued fear memory but not contextual fear memory and sensorimotor gating, our data seemingly accomplished this objective of building early and selective cognitive impairments in an AD-like model of sporadic dementia in rats. Clarifying selective changes in the first stages of AD might enable more specific diagnosis, allowing therapeutic interventions before the emergence of multiple irreversible damages.

Most studies using icv-STZ focused on the investigation of spatial memories, especially because of their dependence from hippocampus, which has been widely described to be compromised in SAD development (Braak and Braak, 1997; Shoham et al., 2003; Grünblatt et al., 2007). Likewise, inhibitory avoidance (IA) performance is also deteriorated at 3–9 months after icv-STZ (1–3 mg/kg) (Knezovic et al., 2015). Contextual fear memories, which are also emotional memories dependent on the hippocampus, have mostly been investigated in transgenic models of AD and the results are still not very clear. Deficits of retention of these memories seems to occur at later stages of AD, between 7 and 9 months in PS1-APP mutant mice (Roy et al., 2016) and only deficits of intra-session acquisition were found earlier, at 4 months of age in AβPPswe/PS1ΔE9 mice (Maroof et al., 2014). However, España et al. (2010) reported an increase in the freezing time of APPSwInd mice aged 12 months, diverging from the other studies. Our data show that the performance of contextual fear memory is not impaired in STZ-treated animals, at least during early evaluation (34–35 days post-treatment), corroborating most studies using transgenic animals.

We also observed an impairment of tone fear memory, an emotional memory which is unaffected by hippocampal lesion (Phillips and LeDoux, 1992) but it is dependent on the striatum (Schenberg et al., 2006; Ferreira et al., 2008): this suggests that after STZ, other brain areas involved in cognitive processes besides the hippocampus, also undergo an early disruption. Interestingly, STZ-icv injected animals showed, 30 days later, a marked increase in p-tau and Aβ peptide in basal ganglia (Santos et al., 2012) corroborating the idea that other forebrain structures and cognitive function related to them are disrupted in this model of SAD. Considering genetic models, impairment of cued fear memories has been described previously in 5XFAD and TgCRND8 mice, which presented less freezing time than wild type during TFC test (Yang et al., 2017).

PPI, on the other hand, is an unlearned cognitive process that does not induce an associative memory between tone pulse and response, but instead a pre-attentional unconscious response (Lichtenberg et al., 2008) involving subcortical areas, such as brainstem nuclei that might also be damaged in AD (Arendt et al., 2015; Hormigo et al., 2017). Although it is known that PPI is compromised in AD (Salem et al., 2011), there is still no data about PPI performance after STZ-icv. Our study showed a lack of alterations in PPI and ASR in STZ-icv as was also observed in APPxPS1 mice aged 9 months (Cheng et al., 2014b). In contrast, another study rTg (tauP301L) 4,510 mice with a phenotype of psychotic AD, reported PPI deficits in animals aged 4–5 months (Koppel et al., 2014). Differences of PPI responses in different AD models could be explained by the anatomic patterns of the neuropathological progress of AD in different types of familial AD (represented by transgenic models) compared to SAD. This may be related to the expression of the different behavioral symptoms in different AD patients, some with depressive symptoms, other with psychotic behavior and others only with cognitive deficits.

The prevention by AEA of some of the cognitive processes disrupted by STZ is consonant to recent studies, which report a growing importance of the role of the endocannabinoid system in the course of AD (Bisogno and Di Marzo, 2008; Fowler et al., 2010; Bedse et al., 2014). This parallels the increasingly recognized therapeutic potential of cannabinoid compounds as potential therapies for many neurodegenerative diseases (Esposito et al., 2006; Carroll et al., 2012; Da Silva et al., 2014). Thus, animals that were administered with cannabidiol (CBD) for 3 weeks after β-amyloid icv injection are protected from the impaired performance in a spatial navigation task and displayed a reduction of the levels of neuroinflammatory mediators (Martín-Moreno et al., 2011). Long-term oral treatment with CBD for 8 months also prevented deficits of social recognition memory in transgenic AβPPSwe/PS1ΔE9 (AβPP × PS1) mice (Cheng et al., 2014c) and using the same model, but treating the animals daily with intraperitoneal injections of CBD for 3 weeks, Cheng et al. (2014a) reported that CBD reversed social and novel object recognition deficits without affecting anxiety-related behaviors. In addition, the presently reported ability of AEA to blunt the enlargement of the brain ventricles suggests an indirect preventive action against neuronal death, which is a well-reported neuroprotective feature of endocannabinoids (Rapino et al., 2017; Vrechi et al., 2018), including AEA (Xu et al., 2017). Further analysis of specific markers of neurodegeneration such as Fluorojade, caspase-3 and Bax, which are increased by icv-STZ (Espinosa et al., 2013; Song et al., 2014; Biswas et al., 2016), would be interesting to better understand the mechanisms behind the neuroprotective role of AEA against the ventricle enlargement.

Most of the studies mentioned above were performed using transgenic models and with intraperitoneal or oral administration of phytocannabinoids. Our study, besides using a model of SAD, tested an endocannabinoid, AEA, through a single icv injection. AEA is a less potent agonist of cannabinoid receptors and did not exert any effect on behavior *per se* as expected, given that all the behavioral procedures were carried out 1 month after the administration of the drugs. AEA was neuroprotective in the deficits of recognition and non-associative emotional memory, but not in associative emotional memory impairment. Although cannabinoid compounds are lipid molecules that can easily cross the brain, icv administration enables a greater control of the concentration of AEA in the brain which is required for an efficient preventive action of the compound. Further studies with other doses of AEA and with drugs that inhibit FAAH, enhancing AEA internal levels, are required for an even more precise evaluation of the relation between AEA levels and AD development. Our results further indicated a tendency for lower CB1R levels only in Veh/STZ. This might be a consequence of the tight interaction between endocannabinoids, CB1R and insulin receptors and their signaling that was previously reported in different systems and brain areas (Kim et al., 2011; Labouèbe et al., 2013; Osmanovic Barilar et al., 2015; Pinheiro et al., 2016). Yet, it is important to remind that AEA also activates CB2R, transient receptor potential vanilloid channels (Rodella et al., 2005) and has membrane and intracellular targets, since it is a hydrophobic molecule that can easily cross lipid membranes (Glaser et al., 2005). Thus, it cannot be ensured that AEA only activated CB1R and it is difficult to determine the pathways operated by AEA to afford its neuroprotective action.

We investigated the levels of CB1R, synaptophysin, SNAP-25 and syntaxin, to verify the existence of a putative synaptic dysfunction in early SAD so that later p-tau alterations that would mediate synaptic changes could be investigated. Synaptophysin and syntaxin were reduced by STZ while SNAP-25 remained unchanged. Notably, AEA only prevented the STZ-induced decrease of syntaxin levels. The brain of AD patients also displays a different alteration of different presynaptic markers, namely a 30% decrease of synaptophysin levels while syntaxin and SNAP-25 were reduced by 10%, probably because each of these presynaptic markers has a different subsynaptic localization and function. Although the present data are indicative of a synaptic dysfunction in this STZ model of SAD, further studies with different synaptic markers will be required to better characterize the pattern of synaptic alterations.

We further tested if the accumulation of hyperphosphorylated tau could be associated with synaptic modifications after icv-STZ. Our results agreed with previous studies that showed an early decrease of p-tau Ser199/202 (Osmanovic Barilar et al., 2015) and did not find differences regarding the levels of p-tau Ser396 between 0.5 and 1 month after STZ injection. Since Veh/STZ and AEA/STZ displayed the same pattern of alterations of p-tau levels it cannot be concluded that these changes are correlated with AEA preventive effect on cognitive deficits.

We also analyzed HSP70 and BAG2 levels and observed that HSP70 levels were only damped in Veh/STZ while all treatments diminished BAG2 levels, with a more pronounced reduction in AEA/Veh and AEA/STZ. Since recent evidence showed that endocannabinoids can be stored in defined reservoirs like the adiposomes and by intracellular trafficking performed by cytosolic carriers such HSP70 and albumin, it is possible that AEA or its metabolites might have been stored and could still be present to modulate the levels of p-tau and BAG2 even long after their administration (Maccarrone et al., 2010). Curiously, another non-selective agonist of CB1R, curcumin (an antioxidant polyphenol) (Witkin et al., 2013) prevented cognitive deficits when administered for 30 consecutive days after icv-STZ injection (Bassani et al., 2017) and when tested *in vitro* curcumin up-regulated BAG2 levels and reduced p-tau levels (Patil et al., 2013). Although these studies reported that levels of BAG2 increased after CB1R activation, this study hints at the possibility that the activation of the endocannabinoid system might interfere with BAG2 levels in a manner dependent on many factors such as the type of cannabinoid administered, the duration of treatment and the physiopathological context. Since HSP70 and BAG2 can also afford neuroprotective actions through mechanisms independent of tau phosphorylation, other players in p-tau metabolism might be involved, such as CHIP, kinases and other forms of phosphorylated tau protein. Their future study might allow understanding the presently observed dissociation between tau phosphorylation and early memory deficits triggered by STZ.

Although the present study provides a first proof of concept for a potential of AEA to control memory dysfunction in SAD, it has several limitations. In fact, we optimized the icv-STZ model in adult rats whereas SAD is prevalent in aged individuals. Thus, further studies should aim at optimizing an icv-STZ model of SAD in aged rats. Also, we only tested male rats, whereas AD is actually prevalent in women, however, female rats are generally not used in studies with STZ because they seem to be resistant to the neurodegenerative effects of STZ, possibly due to the hormonal oscillations of the estral cycle (Bao et al., 2017). Finally, the consolidation of a role of AEA in SAD should require testing different doses of AEA administered both icv and systemically, in an acute manner as well as with different chronic schedules.

Altogether, our results showed, for the first time, that the administration of an endocannabinoid can prevent cognitive, synaptic and histopatological AD-like alterations induced by STZ, thus prompting endocannabinoids as a candidate therapeutic target in AD.

## Author Contributions

JdS-L performed the histological experiments and helped in the surgery procedures and data analysis. DM-S, DC, and TF conceived and planned the study. DM-S, DC, TF, RC, and MA provided the financial support. DM-S, AO, SR, JS-L and PC collected the data. DM-S, AO, SR, JdS-L, PC, RC, MA, DC, and TF analyzed and interpreted the data. DM-S, AO, SR, PC, RC, JdS-L, MA, DC, and TF wrote and reviewed the manuscript.

## Conflict of Interest Statement

The authors declare that the research was conducted in the absence of any commercial or financial relationships that could be construed as a potential conflict of interest.
