# Supplementary figures and images for: Anandamide Effects in a Streptozotocin-Induced Alzheimer’s Disease-Like Sporadic Dementia in Rats
Source: Front Neurosci. 2018 Sep 21;12:653. doi: 10.3389/fnins.2018.00653 (PMC6176656; doi:10.3389/fnins.2018.00653)

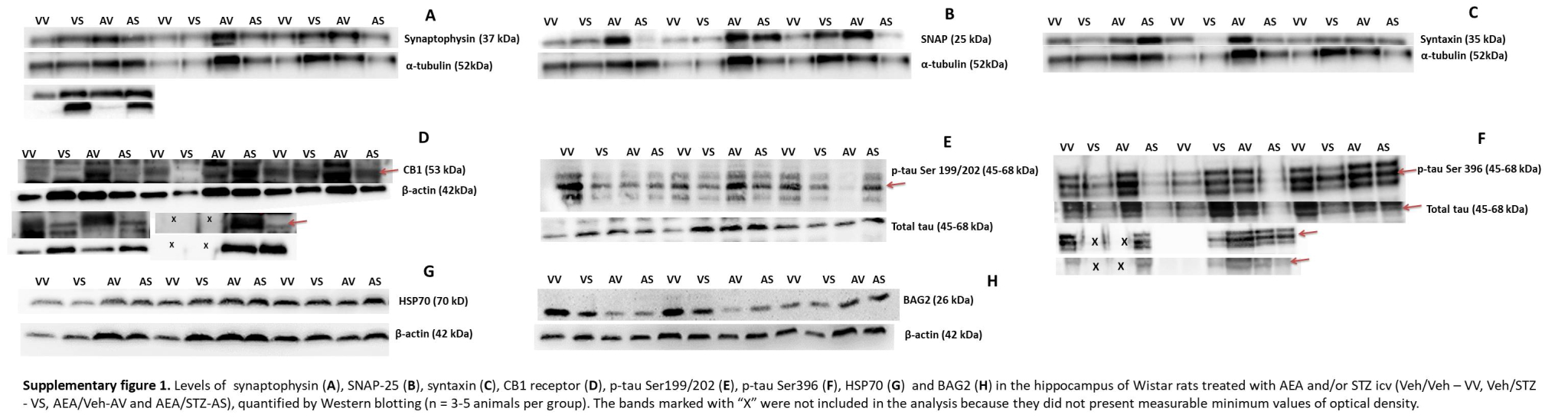

Supplement: Supplementary file 1 [file Image_1.JPEG]
